# The value of data privacy during the COVID-19 pandemic: a new set of survey questions

**DOI:** 10.1186/s42409-022-00037-y

**Published:** 2022-09-05

**Authors:** Susanne Helmschrott, Kartik Anand, Sophie Zhou, Tobias Schmidt

**Affiliations:** Deutsche Bundesbank, Research Centre, Wilhelm-Epstein-Straße 14, 60431 Frankfurt am Main, Germany

**Keywords:** Data privacy, Willingness to pay (WTP), Public health

## Abstract

Contact-tracing smartphone apps that rely on users’ private data have been proposed as important tools in fighting the COVID-19 pandemic. The use of these apps, however, has sparked new debates on the value of data privacy. Several earlier studies have investigated citizens’ willingness to use such apps. We propose a set of questions as a new measurement instrument that goes beyond eliciting acceptance and aims at quantifying users’ willingness to pay (WTP) for data privacy. We assess some aspects of the measurement instrument pertaining to its validity. We find central assumptions of our theoretical model met, suggesting that the instrument serves as a good starting point for measuring WTP. For example, we found a rather low WTP for data privacy in times of a pandemic, with high consent rates to data sharing and a majority of people who would pay amounts of up to 10€ only to not have to share data. Nevertheless, there are several improvements to the instrument possible that should be addressed by future research. We also encourage researchers to field the refined version in larger samples including the offline population.

**Supplementary Information:**

The online version contains supplementary material available at 10.1186/s42409-022-00037-y.

## Introduction

As the largest global health crisis in recent history, the COVID-19 pandemic fundamentally disrupted many aspects of our economic and social life. Digital solutions such as contact-tracing apps that rely on private data have been proposed as important tools in fighting the pandemic, causing a debate on data privacy in Germany, where citizens generally value data privacy highly. Earlier studies in this context have looked into the willingness to accept contact-tracing apps (Altmann et al., 2020; Blom et al., 2021). We add to this literature by developing a new set of survey questions to quantify the willingness to pay (WTP) for data privacy during the COVID-19 pandemic and how the WTP varies with the efficacy of the contact-tracing system. WTP is a well-established concept in economics that has been widely applied to research on privacy. It captures the maximum amount an individual would pay to opt out from their personal data being disclosed (Acquisti et al., 2013; Gopavaram et al., 2021). In this paper, we assess basic quality aspects of the measurement instrument for WTP that was included in the Bundesbank’s Online Panel on Households’ Expectations (BOP-HH).

### The Bundesbank’s Online Panel on Households’ Expectations

The BOP-HH^1^ is a monthly online survey collecting information on individuals’ expectations regarding key economic indicators in Germany. In addition to these core questions, the BOP-HH includes modules on current topics and questions proposed by Bundesbank and external researchers. Since May 2020, questions pertaining to the COVID-19 pandemic have been covered in these modules.

The survey is conducted online by forsa and covers individuals who are at least 16 years old and have used the internet at least once during the previous month. While the survey includes the German online population only, the access panel from which the samples for the BOP-HH are drawn (“forsa.ominet”) is recruited offline from a probability sample. The target sample size for each monthly survey is currently 2500 individuals.

### The value of data privacy

Privacy can be described as the control over and safeguarding of personal information (Westin, 1968). Attitudes towards data privacy vary considerably across countries. In Germany, for example, many citizens share a strong view in favor of greater data protection and privacy.^2^

With the introduction of contact-tracing apps relying on private data in many countries, including Germany, the COVID-19 pandemic sparked a new debate on data privacy. In a cross-country online survey conducted between March and April 2020, Altmann et al. (2020) found a 75%^3^ acceptance rate to install a hypothetical contact-tracing app, with lower acceptance in Germany (70%). In an online survey conducted in June 2020, Blom et al. (2021) found an acceptance rate of 35% to install the “Corona-Warn-App” 4, developed by authorities, among adults in Germany.^5^ While privacy concern consistently shows up as a major determinant of the acceptance rate in these studies, how much individuals value privacy is not explicitly investigated. This research gap motivates us to develop a new measurement instrument to tackle this question.

#### Monetary valuations of privacy

In economics, using surveys to elicit monetary values of nonmarketed goods and services^6^ dates back to the 1940s (Carson, 2012). By revealing the monetary tradeoff each person would make^7^, the results of these surveys provide an estimate of the benefit of such nonmarketed goods in monetary terms and serve as an important input to cost-benefit analyses of public policies.^8^ Privacy is such a nonmarketed good, and many empirical studies have sought to elicit individuals’ monetary value of it. In particular, the literature has focused on two approaches: (i) the minimum monetary incentive individuals are willing to accept in exchange for disclosing their privacy (willingness to accept or WTA), see Huberman et al. (2005) and Hui et al. (2007); and (ii) the maximum amounts that individuals are willing to pay in order to opt out from the obligation to share their private data (WTP); see Beresford et al. (2012), Strahilevitz and Kugler (2016), and Fuller (2019). Theoretically, these two measures reflect different assignments of property rights: the WTA approach sets the status quo as the reference point and treats individuals as entitled to privacy, while under the WTP approach individuals are not entitled to withholding information of public interest. Due to this difference and the resulting “endowment effect” 9, WTA tends to exceed WTP (Carson & Hanemann, 2005; Winegar & Sunstein, 2019).^10^ Both measures are theoretically sound, but the WTA approach is found to consistently elicit high level of protest responses with many respondents refusing to accept any amount or only an infinitely large amount of compensation. WTP thus remains the more reliable and considerably more widely used approach (Perman et al., 2011).

Our new set of questions adds to the existing literature in three ways. First, it goes beyond eliciting the acceptance rate and seeks to quantify the WTP for data privacy. The pandemic has highlighted that individual data privacy can come at a high public cost (loss of lives, costs to the health care system, economic costs, etc.). Quantifying the value of data privacy enables the assessment of such trade-offs for governmental policy. Second, we use different scenarios on the efficacy of data processing to tease out variations in the perception of public health benefits and the risk of privacy loss. Finally, while existing survey evidence mostly originates from the early stage of the pandemic, our study is conducted 1 year later, where the perception of the severity and impact of the pandemic may have changed.

### A simple framework

We consider the problem of a risk-neutral individual (pronoun “she”) who must decide on allowing the state’s public health authority to collect private data from her smartphone. The authority is tasked with implementing a contact-tracing system and advising the government on how to contain the pandemic. Individuals are given the choice to either consent to the data collection or to pay a one-off fee to the government.

An individual *i*’s decision to consent, instead of rejecting data collection, is the result of a cost-benefit analysis. The benefits include an improvement in public health (*H*), which benefits all individuals, and the avoidance of the one-off fee (*F*) to the government. The cost of consenting is the loss of data privacy (*P*_*i*_), where the subscript *i* indicates individual characteristics. We assume that the perceived loss of privacy depends on the distrust for the authority’s digital security (*λ*_*d*, *i*_), while the private benefit of higher public health depends on the individual’s risk profile (*λ*_*r*, *i*_; for example, her exposure to the virus and existing preconditions). An individual would reject data collection as long as her net benefit of consenting, *λ*_*r*, *i*_*H* + *F* − *λ*_*d*, *i*_*P*_*i*_, is negative. The highest fee ${F}_i^{\ast }$ she is willing to pay is one that makes her indifferent between rejecting and consenting.

As an extension to this framework, we introduce the efficacy of the authority in using the collected data (*λ*_*a*_). A more effective contact-tracing system means that the authority can make better use of the data and has a finer picture of individual behaviors. Thus, the private benefit from better public health shifts from *λ*_*r*, *i*_ to *λ*_*r*, *i*_ + *λ*_*a*_. But, at the same time, this also implies a higher perceived loss of privacy so that the perceived loss of privacy shifts from *λ*_*d*, *i*_ to *λ*_*d*, *i*_ + *λ*_*a*_. Thus, the highest fee she is willing to pay for her privacy is:

$${F}_i^{\ast}\equiv \left({\lambda}_{d,i}+{\lambda}_a\right){P}_i-\left({\lambda}_{r,i}+{\lambda}_a\right)H\ .$$

We further note that a higher *λ*_*a*_ should increase ${F}_i^{\ast }$ for individuals who value privacy strongly (*P*_*i*_ > *H*), but decrease ${F}_i^{\ast }$ for those with weaker privacy concerns (*P*_*i*_ < *H*).

## The set of survey questions on the value of data privacy during the COVID-19 pandemic

The objectives of our measurement instrument are a) to examine the WTP for data privacy, which includes the consent rate (share of respondents with ${F}_i^{\ast}\le 0$) and the distribution of ${F}_i^{\ast }$ among respondents who reject data collection (${F}_i^{\ast }>0$), and b) how this varies with the efficacy of the authority’s data processing. To the best of our knowledge, our measure is the first to cover both aspects.

### Implementation of the theoretical framework

We transcribed the framework above into a set of survey questions. Respondents were first presented with a hypothetical scenario in which the German government is planning a contact-tracing system that would allow the Robert-Koch-Institute (RKI)^11^ to assess the spread of COVID-19 in the community. Importantly, relative to the true picture, the assessment by the RKI will be 75% accurate. Based on this assessment, regional or local easing of lockdown measures would be formulated if the situation allows it.

In a first question, respondents were given the choice of either consenting to the collection, use and temporary storage of their smartphone data and COVID-19 test results, or paying a one-off fee to the government. Respondents who did not own smartphones were given the possibility to consider how they would respond if they had access to one.^12^ Those who said they would not consent to providing data were further asked to state the maximum amount they would be willing to pay for opting out. We then posed these questions for an alternative scenario in which the assessment by the RKI is only 50% accurate (question texts see Additional file 1).

We considered four key aspects when designing the set of questions: (1) the framing of the hypothetical scenario, (2) question design, (3) wording and (4) the amount of information to provide.

In line with the concept of WTP, we framed the hypothetical scenario as one where choices are given between consenting to data collection and refusing but paying a fee. We then implemented a two-stage response format: first we ask whether respondents consent to data sharing or not and second, for those who did not consent, we ask to provide the maximum fee they would be willing to pay. We used an open-ended question to elicit the fee instead of offering pre-defined categories to avoid anchoring and bunching effects.

One issue with implementing a two-stage design to measure WTP is that results from the first stage, i.e., the consent rate, are potentially biased. As mentioned in section “Monetary valuations of privacy”, the WTP approach sets the reference point as one where individuals have no entitlement to withholding information of public interest which makes respondents value their personal data less (lack of “endowment effect”). Hence, the consent rates to data sharing are supposedly higher compared to approaches that treat individuals as entitled to privacy (such as WTA or studies measuring consent to data sharing as such). Furthermore, without knowing the size of the one-off fee, respondents’ answers in the first stage depend on what they think the one-off fee is going to be. Depending on whether this biases the consent rate upward or downward, this may either add further bias or reduce the known bias in the consent rate. This bias is only strong, however, if all respondents think of either a very high or very low fee when answering the first stage question. Since this is unlikely, in aggregate, this bias could very well be low. An alternative would be to subdivide the answering category “No, would […] pay a one-off fee” into different fee brackets.^13^ However, as mentioned above, we preferred an open-ended question format since we did not want to frame respondents’ WTP. Another alternative would be eliminating the first stage and directly ask for respondents’ WTP in an open-ended format. In this case, respondents who consent to data collection would be advised to input “zero” as their WTP. However, this would make it impossible to discern between those who would consent from those who do not want to share their data but also do not want to pay for privacy. Furthermore, entering “zero” in case of agreement to data sharing is less intuitive for respondents to understand. Since we wanted to avoid measurement error for our main concept, the WTP, we decided to use a two-stage format.

On the wording of the scenario texts and questions, we made sure to stress that we are presenting a hypothetical situation in order to prompt respondents to detach themselves from the status quo where they are entitled to keep their personal data for themselves at no additional cost (not installing the Corona-Warn-App comes without consequences). To prompt respondents to situate their responses within the hypothetical scenario where they have to pay a fee for protecting their privacy, we repeat wordings like “[p]lease imagine that …” on several occasions in the scenario text. Furthermore, an important choice on wording was whether to present a “tax” or a “fee”. We opted for “fee” since respondents might have diverse attitudes towards taxes, which might interfere with their WTP for data privacy.^14^

Regarding the amount of information given in the question, we aimed at providing respondents with detailed information on the data collection while avoiding a lengthy text. We included the most central information regarding data protection in the text, i.e., which data would be collected, which agency would use the data, where the data would be stored and for how long. At the same time, we did not give details on why the contact-tracing is more or less effective across scenarios. We thought of situations such as the RKI loosing parts of the data collected, not being able to use it properly because of a lack of resources, or prediction models being imperfectly specified for the 50% scenario. In the 75% scenario, the entirety of the data would be used and prediction models would have high accuracy.

We also omitted information that might introduce confounding effects. First, while we made clear that data from the smartphone would be used, we did not describe the exact technical implementation of the system to avoid confusion with the German Corona-Warn-App. Additionally, we did not specify how the government would use the fees. One possibility would have been to suggest that the fees contribute towards financing economic losses stemming from the lockdown. This could, however, confound preferences for privacy with views on the lockdown.

Our choice for a short text certainly has its disadvantages. Since we left details up to respondents’ imagination, they might have understood scenario texts differently. For example, it is possible that some respondents might equate the hypothetical app proposed with the official Corona-Warn-App or they might not understand that a higher efficacy of the track-and-trace system would also yield a higher data loss. Given the short time frames available for questionnaire development in the monthly survey of the BOP-HH, we were not able to conduct a pretest of the instrument. Nevertheless, in our regular assessment^15^ of the questionnaire, only 8% of respondents said they found answering it difficult.

### Assessing quality aspects of the measurement instrument

The survey was completed by 2718 respondents (completion rate: 87%) between April 16 and April 27, 2021.^16^ This corresponds to the later stage of the third wave of infection in Germany, when the average incidence rate varied between 160 and 169 new cases per 100.000 inhabitants and a large-scale lockdown required shops, restaurants, cultural, and sports facilities to remain fully or partially closed (German Federal Government, 2021; RKI, 2021b). In the following, we present analyses assessing basic quality aspects of the measurement instrument, yielding insights into its validity and reliability.

#### Methods

We first examine whether the overall consent rate, as well as the distribution, mean, and median WTP in each efficacy scenario are in line with our model. Second, we look at item-nonresponse to investigate if respondents had difficulties answering our questions or did not want to answer. Third, we assess whether responses are consistent across scenarios. Finally, we examine whether results across subgroups are in line with our expectations.^17^ For the subgroup analyses, we use data from the same survey wave that, according to our model, may influence the respondents’ decisions. The variables used can be grouped into two dimensions: (1) *Trust in and openness towards digital solutions*, captured by several questions on attitudes towards the introduction of a Digital Euro^18^. We expect these questions to mirror attitudes towards the use of a COVID-19 track-and-trace app, since they cover relevant aspects like the willingness to use digital technology, despite potential data privacy concerns. (2) *Individual risk*, measured by the incidence rates at “Landkreis” (i.e., county)–level and differences between age groups, which we use as a proxy for pre-existing health conditions.^19^ To enrich our analysis, we add (3) *labor market affectedness,* measured by employment status. The employment status may serve as proxy variable for being affected by the lockdown, which in turn might influence the WTP for data privacy.

For the subgroup analyses, we estimate the consent rates by means of logistic regressions including only one covariate at a time, using the margins command in Stata 16.0. To test for significant differences between subgroups, we run chi-square test.^20^

Given the high consenting rates, not many respondents provided an answer on the amount they were willing to pay (*N*=340 in the 75% scenario and *N*=528^21^ in the 50% scenario). Due to these low numbers, we could only derive tendencies from the subgroup analyses and hence refrain from these analyses here.

## Results

### Overall results, item non-response, and consistency across scenarios

**Fig. 1:**
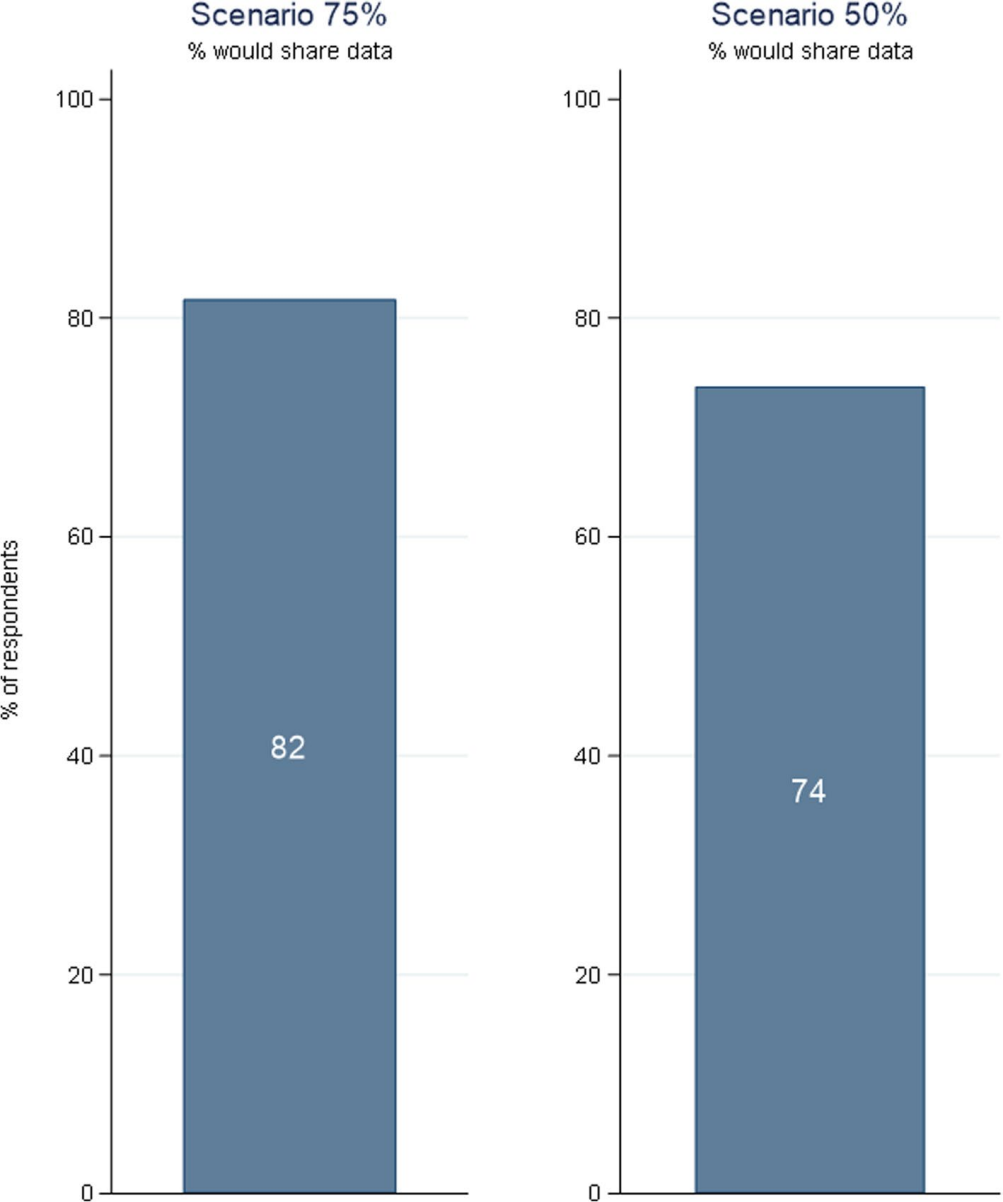
Consent to share data across scenarios*. Note. N*=2667 (75%); *N*=2653 (50%)

Figure 1 shows that in the 75% scenario, 82% of respondents would chose to consent to data sharing, while 18% would pay a one-off fee instead. Faced with the 50% accuracy scenario, the consent rate falls to 74%.^22^

These overall results are in line with our theoretical prediction, which is a first indication towards the instrument’s validity. Even though similar studies found a wide range of acceptance rates for a contact-tracing app among Germans (Blom et al., 2021: 35% vs. Altmann et al., 2020: 70%), we expected high consent rates for several reasons. First, compared to previous studies, the survey was conducted during a high COVID-19 incidence phase and towards the end of a long-lasting lockdown period when citizens were very tired of the situation (Thurm, 2021, March 31). This likely increased the system’s perceived public health benefit. Second, the app is introduced as part of a public program, which assures respondents both of the legitimacy of the data collection and that data will be treated as confidential—contrary to an app run by a private company. Third, compared to using WTA or a design that examines acceptance without (monetary) consequences, the WTP approach tends to overestimate acceptance (see sections “Monetary valuations of privacy” and “Implementation of the theoretical framework”). Finally, the BOP-HH includes the online population only, who might in general be more open to digital solutions than those not using the Internet.^23^

Furthermore, the finding that the consent rate in the 50% scenario is significantly lower is also in line with our expectations since a lower accuracy may reduce the public health benefit. Since it also reduces the privacy loss, it decreases ${F}_i^{\ast }$ for those with strong privacy concerns. Thus, some respondents who consented in the 75% scenario could now refuse data sharing.

Those refusing were on average willing to pay a maximum of 44€ in the 75% scenario, compared to 30€ in the 50% scenario. The median value, however, is identical in both scenarios (10€), and the distribution of values is also very similar (see below), indicating that the differences in the mean values may be due to outlier values remaining after trimming^24^. This suggests that contrary to our assumption in the model, respondents may not equate a higher efficacy of the contact-tracing system with a greater loss of data privacy. Hence, the instrument should be improved by making this connection clearer in the scenario texts.

**Fig. 2:**
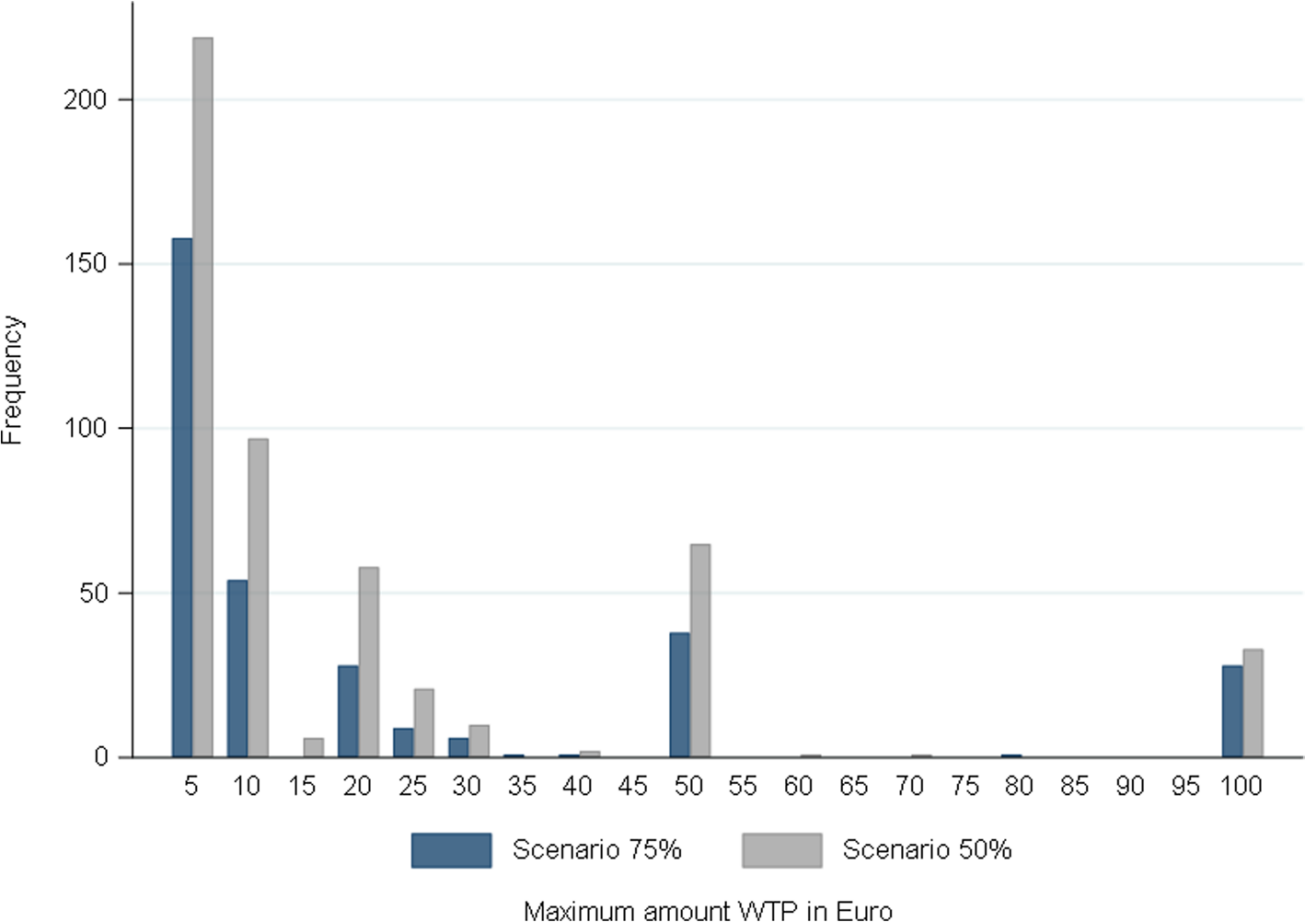
Distribution of the maximum amount WTP across scenarios*. Note. N*=324 for 1–100 EUR and *N*=16 for >100 EUR (scenario 75%); *N*=513 for 1–100 EUR and *N*=15 for >100 EUR (scenario 50%). Values above 100€ were only provided by *N*=16 (scenario 75%) and *N*=15 (scenario 50%) with mainly rounded values such as 250, 300, and 400. The (trimmed) maximum amount 500€ was given by *N*=8 in scenario 75% and *N*=4 in scenario 50%

Figure 2 shows that in both scenarios, the distribution of the Euro-amounts is very left-skewed, with most respondents providing values of up to 10€ and about a third of respondents only 1€ (Additional file 2, Fig. S1).

Given the concept measured, these findings point to the instrument’s validity, too. Since under the WTP approach respondents are not entitled to withhold personal data that is important for public health, they attach a rather low value to it (cf. endowment effect). Accordingly, a low mean WTP with a left-skewed distribution is in line with our expectations.

Looking at the distribution, we can furthermore see that choosing an open-ended question format over a categorical question format could not fully avoid a bunching of responses. Indeed, there are visible clustering effects at rounded values such as 5, 10, 20, or 100 Euros.^25^ Since we do not know whether respondents rounded their “true” WTP up or down, this somewhat decreases the precision of our measurement.

Item-nonresponse to the overall consent to data sharing was low in both scenarios (<2%), whereas about every tenth respondent who did not consent to data sharing gave no answer regarding the amount he or she was willing to pay (13% in scenario 75%; 10% in scenario 50%). There are several potential reasons for this substantial share of item-nonresponse. Some respondents might have had difficulties assigning a value to data privacy. Others might have opted to not answer because of the hypothetical nature of the question. Indeed, being asked to provide a monetary value for the protection of their personal data, which in reality they get free of charge might have left some respondents confused or irritated. Given that we also could not avoid bunching by using an open-ended format, future research could address whether a closed categorical answering format would have yielded similar answers while at the same time allowing for lower item-nonresponse by providing some orientation for respondents. When using an open-ended format, researchers should impute missing values in order to reduce potential bias in the maximum amount respondents are willing to pay for data privacy. Moreover, future research could examine whether stressing the hypothetical nature of the contact-tracing system even more reduces or increases item non-response.

Given that we could not run the questions on another sample, the possibilities of assessing the measurement instrument’s reliability are very limited. Nevertheless, examining the consistency across scenarios allows for some insights here. Since we varied only one parameter among many that influence the individual’s WTP for data privacy, we expect that a large majority of respondents were consistent in either consenting or rejecting in both scenarios. Indeed, 88% consent and 92% reject in both scenarios. However, there are also a few cases (8%) that would be willing to pay in the 50% scenario only, which cannot be explained by our model. This finding might serve as further indication that (some) respondents did not equate a lower effectiveness of the track-and-trace system with a decreased loss in data privacy.

### Subgroup analyses

**Fig. 3:**
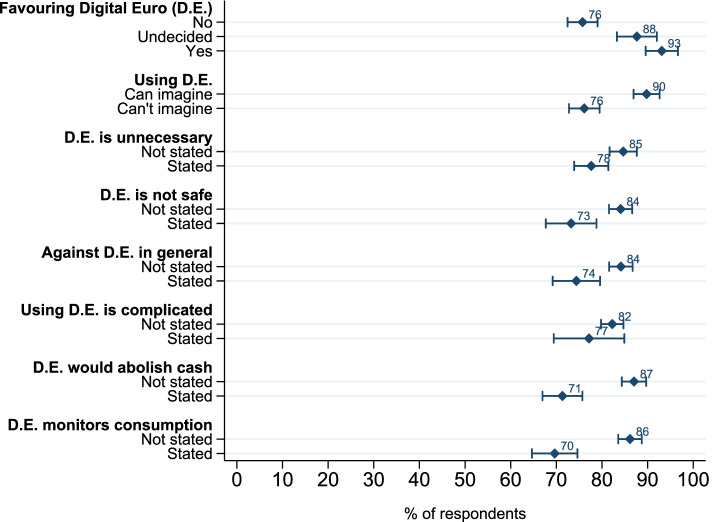
Predicted consent rates to data sharing by trust in and openness towards digital solutions — scenario 75%. *Note. N* is between 2654 and 2664. Error bars represent 95% CI. All predicted consent rates per subcategories within each variable are significantly different from each other (*p*<0.01), aside from “Favouring D.E.” “undecided” and “yes”, and “Using D.E. is complicated” “stated” and “not stated”

As shown in Fig. 3 and Figure S2 (Figure S2 see Additional file 2), we find a strong relationship between respondents’ *trust in and openness towards digital solutions* and their WTP for data privacy across scenarios. Those who are not in favor of the introduction of a Digital Euro and those who cannot imagine using it are predicted to be significantly less likely to consent to data sharing. The predicted differences between consenting and refusing are particularly pronounced among respondents who mentioned data privacy issues (i.e., “[…] monitors consumption”, “[…] is not safe”) as reasons to not support implementing a Digital Euro. This reflects the prediction of our model that a stronger distrust to the authority’s digital security (*λ*_*d*, *i*_) yields lower consenting rates, which is a further indication towards the instrument’s validity.

**Fig. 4:**
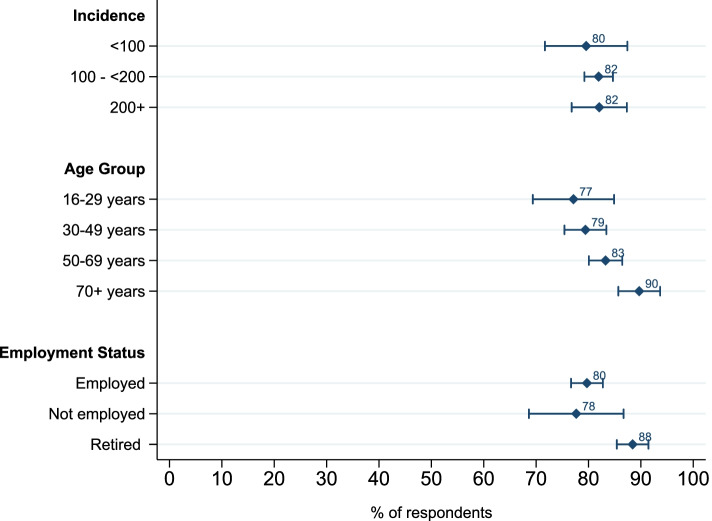
Predicted consent rates to data sharing by individual risk and labor market affectedness — scenario 75%*. Note. N*=2667 for incidence, age, and employment status. Error bars represent 95% CI. Predicted significant differences: *p*<0.01: age groups “16–29 years” vs. “70+ years” and “30–49 years” vs. “70+ years”, “employed” vs. “retired”. *p*<0.05: age group “50–69 years” vs. “70+ years”, “not employed” vs. “retired”

The picture is more mixed when we examine the predicted consenting rates according to *individual risk and labor market affectedness* (Fig. 4). From our model, we would have expected that the higher the *individual risk* is, the higher the public health benefit from consenting is, and hence, the higher the consenting rates would be. We do find this expectation satisfied when looking at the predicted consenting rate according to age, which is significantly higher among the oldest, and hence most vulnerable to a severe COVID-19 infection. Nevertheless, we do not see significant differences in the predicted consent rates according to the counties’ incidence rates. Furthermore, there are no significant differences between subgroups regarding *employment status* except the retired being significantly more likely to consent than other groups, indicating overlaying effects of age and employment. These findings are similar for the 50% scenario (Additional file 2, Fig. S3).

## Discussion and conclusion

In this article, we address basic quality aspects of our set of questions on the value of data privacy during the COVID-19 pandemic. Overall, we find the instrument able to capture the underlying concept well. For example, we found a rather low WTP for data privacy in times of a pandemic, with high consent rates for data sharing and a majority of refusers who would pay amounts of up to 10€ only. Furthermore, we found a strong relationship between consent rates and the trust in and openness towards digital solutions. In addition, consent rates decrease with the lower accuracy of the contact-tracing system. Hence, we consider our instrument to be a good starting point for measuring the value of data privacy using a WTP framework. Nevertheless, some shortcomings of the instrument should be addressed in future research: First, we did not find differences in the median amounts those rejecting data sharing would pay, indicating that the scenario text should explain the differences between the 75% and the 50% scenario better. Notably, the connection between a higher efficacy of the contact-tracing system and a greater loss of data privacy should be made clearer. A factorial survey experiment which varies both the public health benefit and the degree of loss in data privacy of the contacttracing system could further disentangle the influence of these different components on the formation of WTP for privacy. Second, we found an elevated share of item-nonresponse regarding the amount that those refusing to share their data would be willing to pay for protecting it. One explanation for this is that some respondents had difficulties figuring out which amount they consider to be appropriate. Hence, future research could address whether using a closed categorical answering format to measure WTP would be useful by providing some orientation on the range of potential answers for respondents. Although we can only speculate about further reasons for item-nonresponse here, it is also possible that respondents were confused or irritated by the scenario in which they are forced to pay a fee for protecting their personal data while in reality they can protect their data at no cost by not installing Germany’s official Corona-Warn-App. Thus, researchers could examine whether stressing the hypothetical nature of the scenario even more explicitly and stating that the contact-tracing app should not be confused with the Corona-Warn-App has an impact on the results. In addition, further research on the advantages and disadvantages of a two-stage vs. one-stage design would be beneficial.

Our analysis of the instrument’s quality has some limitations. First, we had to omit subgroup analyses on the amount of WTP due to a low number of respondents who chose to refuse data sharing. Second, due to space constraints in the BOP-HH, we had only a limited number of covariates at our disposal. For example, it would have been useful to include the respondents’ at-risk health conditions and the frequency of social contacts they had to refine the measurement of individual risk patterns. Finally, the BOP-HH covers the online population^26^ only. Given that we can assume that the offline population differs from the online population with regard to their WTP for data privacy during the COVID-19 pandemic, this limits the generalizability of our findings.^27^ Given these limitations, the present study of the instrument’s quality is rather explorative. To allow for a more thorough assessment, we encourage researchers to run a refined measurement instrument in surveys representative for the general population with larger sample sizes and additional covariates.

## Supplementary Information

**Additional file 1.** Questionnaire.

**Additional file 2.** Additional figures.

## Data Availability

The data of the Bundesbank Online Panel on Households’ Expectations (BOP-HH) can be made available as Scientific Use Files (SUF) upon application at the Bundesbank’s Research Data and Service Centre (RDSC) using the following link: https://www.bundesbank.de/en/bundesbank/research/rdsc/data-access/data-access-617236. The COVID-19 incidence rates at district level published by the Robert-Koch-Institut can be downloaded from: https://www.rki.de/DE/Content/InfAZ/N/Neuartiges_Coronavirus/Daten/Fallzahlen_Kum_Tab.html/. Daily data on COVID-19 cases at county level is also accessible through: https://npgeo-corona-npgeo-de.hub.arcgis.com/. Variables not included in the standard SUF are the ID of the counties (“Kreiskennziffern”), which was used to merge the COVID-19 incidence data. These data can only be accessed on-site at the Bundesbank’s RDSC. Furthermore, information on the sample design cannot be retrieved from the SUF.

## Footnotes

1 Additonal information on the survey are available on the Bundesbank website: https://www.bundesbank.de/en/bundesbank/research/survey-on-consumer-expectations/survey-on-consumer-expectations-848330. A description of the methodology is available in Beckmann and Schmidt (2020).

2 As one example, a survey by the European Union Agency for Fundamental Rights in 2020 found that people in Germany have one of the lowest willingness to share private information such as facial profiles amongst all EU citizens (European Union Agency for Fundamental Rights, 2020).

3 The sample included respondents from the UK, Germany, Italy, France, and the USA (Altmann et al., 2020).

4 For more information on the app, see Deutsche Bundesregierung (2022).

5 These widely varying results could stem from methodological differences such as differences in sample selection, timing of the surveys, and questionnaire design. For example, Blom et al. (2021) implemented their study in the German Internet Panel (GIP), a probability-based online panel that includes the offline population. Altmann et al. (2020) fielded their questions in non-probability-based online access panels and reran their analysis for Germany in an offline-recruited online panel with similar results (see Multimedia Additional file 1; accessible on https://www.ncbi.nlm.nih.gov/pmc/articles/PMC7458659/#app1). Both sample types used by Altmann et al. (2020) exclude the offline population, while they are included in the GIP. Given that the offline population is less tech savy, reservations towards data sharing via a smartphone app might be more widespread in the sample of the GIP. Some other studies of willingness to install and use contact-tracing apps include Kaptchuk et al. (2020), Trang et al. (2020) (Germany), Utz et al. (2021) (China, Germany, and the USA), and Lewandowsky et al. (2021) (UK). Overall, these studies tend to find high rates of willingness to install in China, the UK, and the USA, while lower rates in Germany.

6 These are goods and services that are not bought or sold in the marketplace and for which no market price is available.

7 What is of interest here is thus behavior (observable) rather than the underlying attitudes (unobservable).

8 Survey-based valuation studies have been used to assess the benefit of environmental (e.g., the recreation value of national parks), health (e.g., the value of increased ambulance services), and transportation policies (e.g., alternative fuel vehicles), see Carson and Hanemann (2005).

9 The endowment effect describes the finding that people assign a higher value to an object if they own it than if they do not own it (Thaler, 1980).

10 In terms of the acceptance rate for a contact-tracing app, the WTP approach elicits a higher acceptance rate (i.e. a lower opt-out rate) and a lower monetary value, compared to WTA (see also section “Implementation of the theoretical framework”).

11 The Robert Koch Institute is the government’s central scientific institution in the field of biomedicine. Some of its main tasks are the identification, surveillance, and prevention of infectious diseases and advising policy-makers on health issues (RKI, 2021a).

12 This allowed us to identify respondents for who the questions practically were not applicable while at the same time getting an indication on their consent to data sharing under the WTP approach. Due to the non-applicability of the situation to non-smartphone users, they were not presented with the subsequent questions (i.e., “filtered out”).

13 E.g., “Yes, I would consent […]”, “No, I would […] pay a one-off fee of 1-10€”, “No, I would […] pay a one-off fee of 11-50€”, “No, I would […] pay a one-off fee of 51-100€”, etc.

14 In addition, taxes are usually measured as percentage (i.e., of the income), so we would have needed to ask for the change in their tax rate they would be willing to accept to keep their data private. Thus, in order to avoid measurement error, we decided to employ a “fee” which is measured in Euro. We added “one-off” fee to stress that it would be a single contribution.

15 We regularly ask respondents how interesting and difficult they found the questionnaire and whether it was too long.

16 Data was weighted using untrimmed post-stratification weights, which calibrate the survey data to population totals according to region, age, gender, and education (Beckmann & Schmidt, 2020). In order to remove outlier values, data on the maximum amount respondents were willing to pay was trimmed to two times the standard deviation of the mean.

17 Analyses regarding the consent rate per subgroup for the 75% scenario exclude non-smartphone users since they are likely to differ from smartphone users regarding their attitudes towards central concepts measured here, such as data privacy and the use of digital technologies. Per default, they are not part of the subsequent analyses because they were not presented with the respective questions in the questionnaire.

18 Respondents were asked whether they were in favor of introducing a Digital Euro, for the reasons why if they were not in favor and whether they can imagine using the Digital Euro (see Additional file 1).

19 We assume that respondents were more or less aware of their county-level incidence rates since the COVID-19 regulations imposed at that level depended on them (German Federal Government (2021). Age may interfere in various ways with our research interest, such as different age groups responding differently to the government authority, app use, and data sharing. Nevertheless, we believe that in the context of the COVID-19 pandemic, typically with age increasing at-risk-health conditions such as high blood pressure and the resulting vulnerability towards the disease are decisive factors for differences in the willingness to support a system containing the spread of the pandemic.

20 This type of analysis was also used by Blom et al. (2021). To improve variance estimation, we reran the analyses taking the complex sample design into account, using Taylor Series Linearisation. We observed only minor differences in the results of the significance tests which did not have an impact on any of our conclusions.

21 Both trimmed and unweighted counts.

22 Only *N*=5 or 0.2% of the respondents said they did not have a smartphone but would choose to consent and *N*=10 or 0.4% stated they did not have a smartphone but would not consent. Both respondent groups as well as “Don’t know” and “No answer” categories were coded as missing values here.

23 For example, as a Special Eurobarometer Report from 2017 points out: “76% who use Internet everyday say the impact of [digital] … technologies on their quality of life has been positive, compared to 38% who never use the Internet” (European Commission, 2021).

24 Only very few outlier values had to be excluded. For example, in the 75% scenario three times 1000€, and once 5000€ were given. This indicates that respondents took answering the questions seriously.

25 Due to reasons of confidentiality, results are not displayed in detail in the Additional file 2.

26 In the BOP-HH, onliners are defined as having used the Internet at least once during the previous month (see section "The Bundesbank’s Online Panel on Households’ Expectations").

27 For example, the elderly, who are more likely to be offliners (DESTATIS, 2022), are more at risk of getting seriously ill from COVID and hence might be more willing to share their data for fighting the pandemic. However, the share of offliners is small in Germany (e.g., in 2021 only 9% of those living in Germany aged 14+ say they never used the internet; Initiative D21 e.V, 2022), limiting the impact of the exclusion of this group on the overall results.
