# Supplementary material for: The value of data privacy during the COVID-19 pandemic: a new set of survey questions
Source: Meas Instrum Soc Sci. 2022 Sep 5;4(1):10. doi: 10.1186/s42409-022-00037-y (PMC9444084; doi:10.1186/s42409-022-00037-y)
Supplement: Supplementary file 1 — Additional file 1. Questionnaire. [file 42409_2022_37_MOESM1_ESM.pdf]

Additional file 1. Questionnaire

English question texts

Questions on the value of data privacy

Q1

Categories 3 and 4 are shown as soft prompt to respondents
The next questions are about routes out of the coronavirus pandemic.<br>Imagine that the German Federal Government is planning to introduce a contact tracing system similar to that in Israel and South Korea, which would enable the Robert Koch Institute (RKI) to better understand the transmission of COVID-19. Where applicable, this could result in a regional or local easing of the lockdown measures being allowed.<br>Please assume that in <u>75% of cases</u> the system would provide an accurate assessment of the spread of COVID-19 among the German population.<br>For the tracing to work, the RKI would have to collect personal data from members of the German public (<u>name, address, mobile phone number</u>), link these data with <u>GPS/location data</u> from mobile phones as well as <u>COVID-19 test results</u> and save them on a <u>secure data server</u> in Germany for <u>three months</u>.<br>Now please assume that you would be given the choice by the German government of either:<br>a) consenting to the collection, linking and temporary storage of your data (name, address, mobile phone number, GPS/location data, COVID-19 test results);<br>b) or refusing consent to this and instead paying a one-off fee to the German government.<br>**QUESTION:** Would you consent to the use of your data or refuse consent to this and instead pay a one-off fee to the German government?<br>1 Yes, I would consent to the collection, linking and temporary storage of my data (name, address, mobile phone number, GPS/location data, COVID-19 test results).<br>2 No, I would refuse consent to this and would instead pay a one-off fee to the German government.<br>3 I do not own a smartphone that can collect GPS/location data but would consent to the use of my data if I had one.<br>4 I do not own a smartphone that can collect GPS/location data but would refuse consent to the use of my data if I had one.

Q2

Input filter: if Q1 = 2
Range of valid values: 1 to 999,999
**QUESTION:** What is the maximum amount you would be willing to pay in order for your data not to be used?<br><br>**Note:** Please enter a figure in euro without decimal places.<br><br>[Input field] euro

Q3

Input filter: if Q1 = 1 | 2
Now assume that the contact tracing system only gives an accurate assessment of the spread of COVID-19 in <u>50% of cases</u> (instead of 75% as in the previous question).<br><br>**QUESTION:** Would you consent to the use of your data or refuse consent to this and instead pay a one-off fee to the German government?<br><br>1 Yes, I would consent to the collection, linking and temporary storage of my data (name, address, mobile phone number, GPS/location data, COVID-19 test results).<br>2 No, I would refuse consent to this and would instead pay a one-off fee to the German government.

Q4

Input filter: if Q3 = 2
Range of valid values: 1 to 999,999
**QUESTION:** In this case, what is the maximum amount you would be willing to pay in order for your data not to be used?<br><br>**Note:** Please enter a figure in euro without decimal places.<br><br>[Input field] euro

Questions on the Digital Euro

Q1

<table>
<tr><td>Definition digital euro shown in scroll-over infoboxes of Q1-Q5:

“A digital euro would be an electronic form of central bank money. Central bank money is money that can only be created by the central bank. Today, central bank money mainly exists in the form of cash, which the central bank brings into circulation. A digital euro would enable everyone to pay in cashless form with central bank money.

A digital euro is not the same as a crypto-asset, such as bitcoin. A digital euro would be protected and regulated by the European Central Bank. It would also be default-free as a central bank can never go bankrupt. Crypto-assets have so far been issued and traded without any state oversight. This means that there is no institution ensuring values remain stable and, as a result, crypto-assets – unlike digital euro – may be subject to sharp, unexplained fluctuations in value”.</td></tr>
<tr><td>Together with other European institutions, the Bundesbank is considering implementing a digital euro(i).

QUESTION: Had you ever heard or read anything about digital euro(i) prior to this survey?

1 = Yes, I had already heard/read about digital euro.
2 = No, so far I have only heard/read about crypto-assets (e.g. bitcoin).
3 = No, I have not heard/read about digital euro or crypto-assets.</td></tr>
</table>

Q2

<table>
<tr><td>QUESTION: To what extent would you be in favour of implementing a digital euro(i)?

1 = Definitely not in favour
2 = Generally not in favour
3 = Undecided
4 = Generally in favour
5 = Definitely in favour</td></tr>
</table>

Q3

<table>
<tr><td>Input filter: if Q2 = 1 | 2</td></tr>
<tr><td>Allow for multiple answers</td></tr>
<tr><td>Order of categories is generated randomly for each respondent.</td></tr>
<tr><td>QUESTION: For what reasons would you not be in favour of implementing a digital euro(i)?

1 = Item selected
2 = Item not selected

a I find the types of payment already available sufficient.
b I don’t think a digital euro would be secure.
c I am against all forms of digital money.
d It would be too complicated for me to switch to using digital euro.
e I don’t think I would be able to use digital euro everywhere.
f Digital euro would be the first step towards getting rid of cash.
g Digital euro would enable my purchasing habits to be monitored.
h Other (please specify): [Input field]</td></tr>
</table>

Q4

Input filter: if Q2 = 4 | 5
Allow for multiple answers
Order of categories is generated randomly for each respondent.

QUESTION: For what reasons would you be in favour of implementing a digital euro(i)?

1 = Item selected

2 = Item not selected

a	A digital euro would be a good alternative to existing cashless payment instruments.
b	A digital euro would be a good alternative to cash.
c	A digital euro would be a good alternative to commercial payment initiatives (e.g. PayPal).
d	A digital euro would strengthen the international role of the euro.
e	A digital euro is part of a digitalised society.
f	I think a digital euro would be secure.
g	I would trust digital euro more than the existing crypto-assets.
h	Other (please specify): [Input field]

Q5

QUESTION: Which of the following statements regarding the use of a digital euro(i) applies to you?

1 = I can generally imagine using digital euro.

2 = I can generally imagine using both digital euro and crypto-assets.

3 = I cannot imagine using digital euro.

German question texts

Questions on the value of data privacy

Q1

Categories 3 and 4 are shown as soft prompt to respondents
In den nächsten Fragen geht es um Wege aus der Corona-Pandemie.<br><br>Stellen Sie sich bitte einmal vor, die deutsche Bundesregierung plane die Einführung eines Systems zur Nachverfolgung von Kontakten, welches es dem Robert-Koch-Institut (RKI) ermöglichen würde, die Übertragung von Covid-19 besser nachzuvollziehen (ähnlich wie in Israel und Südkorea). Als Ergebnis könnten gegebenenfalls regionale oder lokale Lockerungen der Lockdown-Maßnahmen erlaubt werden.<br><br>Nehmen Sie bitte an, das System würde in 75% der Fälle eine richtige Einschätzung der Ausbreitung von Covid-19 in der Deutschen Bevölkerung abgeben.<br><br>Damit die Nachverfolgung funktioniert, müssten vom RKI personenbezogene Daten der Bürger in Deutschland (Name, Adresse, Mobilfunknummer) gesammelt werden, diese mit den GPS/Ortsdaten der Mobilfunktelefone sowie den Covid-19 Testergebnissen verknüpft und auf einem sicheren Datenserver in Deutschland für drei Monate gespeichert werden.<br><br>Nehmen Sie nun an, dass Sie von der deutschen Bundesregierung vor die Wahl gestellt würden, entweder:<br><br>a) Der Erhebung, Verknüpfung und vorrübergehenden Speicherung Ihrer Daten (Name, Adresse, Mobilfunknummer, GPS/Ortsdaten, Covid-19 Testergebnisse) zuzustimmen<br>b) Dies abzulehnen und stattdessen eine einmalige Abgabe an die Bundesregierung zu bezahlen.<br><br>**Frage:** Würden Sie der Verwendung Ihrer Daten zustimmen oder dies ablehnen und stattdessen eine einmalige Abgabe an die Bundesregierung zahlen?<br><br>1 Ja, ich würde der Erhebung, Verknüpfung und vorrübergehenden Speicherung meiner Daten (Name, Adresse, Mobilfunknummer, GPS/Ortsdaten, Covid-19 Testergebnisse) zustimmen<br>2 Nein, ich würde dies ablehnen und stattdessen eine einmalige Abgabe an die Bundesregierung bezahlen.<br>3 Ich habe kein Smartphone, welches meine GPS/Ortsdaten sammeln kann, würde aber der Verwendung meiner Daten zustimmen, wenn ich eines hätte.<br>4 Ich habe kein Smartphone, welches meine GPS/Ortsdaten sammeln kann, würde aber die Verwendung meiner Daten ablehnen, wenn ich eines hätte.

Q2

Input Filter: If Q1 = 2
Range of valid values: 1 bis 999.999
**FRAGE:** Welchen Betrag wären Sie maximal bereit zu zahlen, damit ihre Daten nicht verwendet würden?<br><br>**Hinweis:** Bitte geben Sie einen Betrag in Euro ohne Nachkommastellen an.<br><br>[Input field] Euro

Q3

Input filter: if Q1 = 1 | 2
Und nun nehmen Sie bitte an, dass das System zur Nachverfolgung von Kontakten in nur 50% der Fälle (statt wie in der vorherigen Frage 75%) eine richtige Einschätzung der Ausbreitung von Covid-19 in der deutschen Bevölkerung abgibt.<br><br>**Frage:** Würden Sie der Verwendung Ihrer Daten zustimmen oder dies ablehnen und stattdessen eine einmalige Abgabe an die Bundesregierung zahlen?<br><br>1 Ja, ich würde der Erhebung, Verknüpfung und vorrübergehenden Speicherung meiner Daten (Name, Adresse, Mobilfunknummer, GPS/Ortsdaten, Covid-19 Testergebnisse) zustimmen<br>2 Nein, ich würde dies ablehnen und stattdessen eine einmalige Abgabe an die Bundesregierung bezahlen.

Q4

Input Filter: If Q3 = 2
Range of valid values: 1 to 999.999
**FRAGE:** Welchen Betrag wären Sie in diesem Fall maximal bereit zu zahlen, damit ihre Daten nicht verwendet würden?<br><br>**Hinweis:** Bitte geben Sie einen Betrag in Euro ohne Nachkommastellen an.<br><br>[Input field] Euro

Questions on Digital Euro

Q1

Definition Digitaler Euro, die in scroll-over Infoboxen bei den Fragen Q1-Q5 gezeigt wird:

Ein **Digitaler Euro** wäre eine **elektronische Form von Zentralbankgeld**. Zentralbankgeld ist Geld, das nur von der Zentralbank geschaffen werden kann. Heutzutage existiert das Zentralbankgeld vor allem in Form von Bargeld, welches die Zentralbank in Umlauf gibt. Ein Digitaler Euro würde es jeder/m ermöglichen, mit Zentralbankgeld in unbarer Form zu bezahlen.

Ein Digitaler Euro ist **nicht gleichzusetzen mit Krypto-Assets**, wie z.B. Bitcoin. Ein Digitaler Euro wäre **von der Europäischen Zentralbank geschützt** und **reguliert**. Er wäre zudem ausfallsicher, da eine Zentralbank nicht bankrottgehen kann. Krypto-Assets werden bislang **ohne jede staatliche Kontrolle** ausgegeben und gehandelt. Das bedeutet, dass es keine für Wertstabilität sorgende Institution gibt und in der Folge Krypto-Assets im Gegensatz zu einem Digitalen Euro teils großen und nicht erklärbaren Wertschwankungen unterliegen.

Die Deutsche Bundesbank prüft gemeinsam mit anderen europäischen Zentralbanken, ob ein **Digitaler Euro**(i) eingeführt werden soll.

FRAGE: Haben Sie bereits vor dieser Befragung schon einmal vom Digitalen Euro(i) gehört oder darüber etwas gelesen?

1 = Ja, ich habe bereits vom Digitalen Euro gehört/gelesen.
2 = Nein, ich habe bisher nur von Krypto-Assets (z.B. Bitcoin) gehört/gelesen.
3 = Nein, ich habe weder vom Digitalen Euro noch von Krypto-Assets gehört/gelesen.

Q2

FRAGE: Inwiefern würden Sie die Einführung eines solchen Digitalen Euros(i) befürworten?

1 = Überhaupt nicht befürworten
2 = Eher nicht befürworten
3 = Unentschieden
4 = Eher befürworten
5 = Stark befürworten

Q3

Input filter: if Q2 = 1 | 2

Allow for multiple answers

Order of categories is generated randomly for each respondent.

FRAGE: Aus welchen Gründen würden Sie die Einführung des Digitalen Euros(i) nicht befürworten?

1 = item selected

2 = item not selected

- a Die bisherigen Zahlungsarten reichen mir aus.
- b Ich halte einen Digitalen Euro für unsicher.
- c Ich lehne digitales Geld generell ab.
- d Der Umgang mit dem Digitalem Euro wäre mir zu kompliziert.
- e Ich denke, ich könnte den Digitalen Euro nicht überall verwenden.
- f Ein Digitaler Euro wäre der Beginn der Abschaffung des Bargeldes.
- g Der Digitale Euro überwacht mein Konsumverhalten.
- h Sonstiges, und zwar: [Input field]

Q4

Input filter: If Q2 = 4 | 5
Allow for multiple answers
Order of categories is generated randomly for each respondent.
**FRAGE:** Aus welchen Gründen würden Sie die Einführung des Digitalen Euros(i) befürworten?<br>1 = item selected<br>2 = item not selected<br>a Ein Digitaler Euro wäre eine gute Alternative zu existierenden bargeldlosen Zahlungsinstrumenten.<br>b Ein Digitaler Euro wäre eine gute Alternative zum Bargeld.<br>c Ein Digitaler Euro wäre eine gute Alternative zu kommerziellen Zahlungsinitiativen (z.B. PayPal).<br>d Ein Digitaler Euro würde die internationale Rolle der Euro-Währung stärken.<br>e Ein Digitaler Euro gehört zu einer digitalisierten Gesellschaft.<br>f Ich würde einen Digitalen Euro für sicher halten.<br>g Ich würde dem Digitalen Euro mehr vertrauen als den existierenden Krypto-Assets.<br>h Sonstiges, und zwar: [Input field]

Q5

**FRAGE:** Welche der folgenden Aussagen bezüglich der Nutzung eines Digitalen Euros(i) trifft auf Sie persönlich zu?<br>1 = Ich kann mir grundsätzlich vorstellen, Digitale Euros zu nutzen.<br>2 = Ich kann mir grundsätzlich vorstellen, sowohl Digitale Euros als auch Krypto-Assets zu nutzen.<br>3 = Ich kann mir überhaupt nicht vorstellen, Digitale Euros zu nutzen.
