# Supplementary material for: The value of data privacy during the COVID-19 pandemic: a new set of survey questions
Source: Meas Instrum Soc Sci. 2022 Sep 5;4(1):10. doi: 10.1186/s42409-022-00037-y (PMC9444084; doi:10.1186/s42409-022-00037-y)
Supplement: Supplementary file 2 — Additional file 2. Additional figures. [file 42409_2022_37_MOESM2_ESM.pdf]

Additional file 2. Additional Figures

Fig. S1

Maximum amount WTP in categories

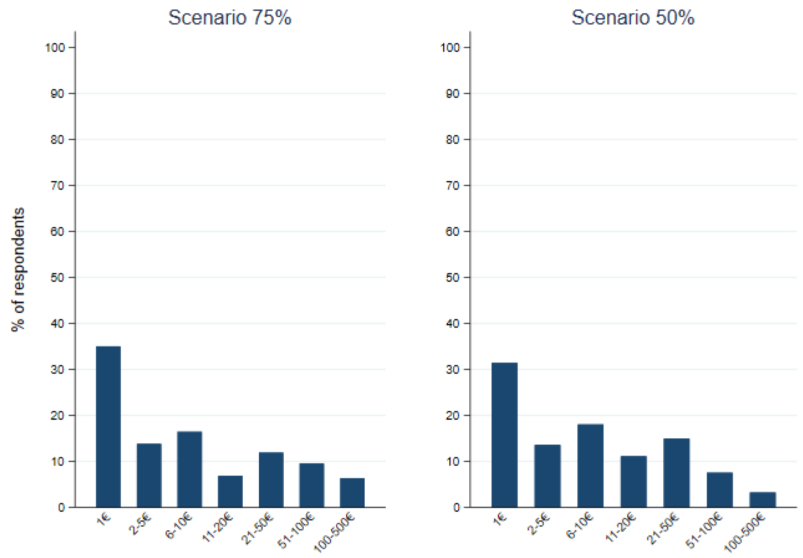

Note. N=340 (75%); *N*=528 (50%).

Fig. S2

Predicted consent rates to share data by trust in and openness towards digital solutions – scenario 50%

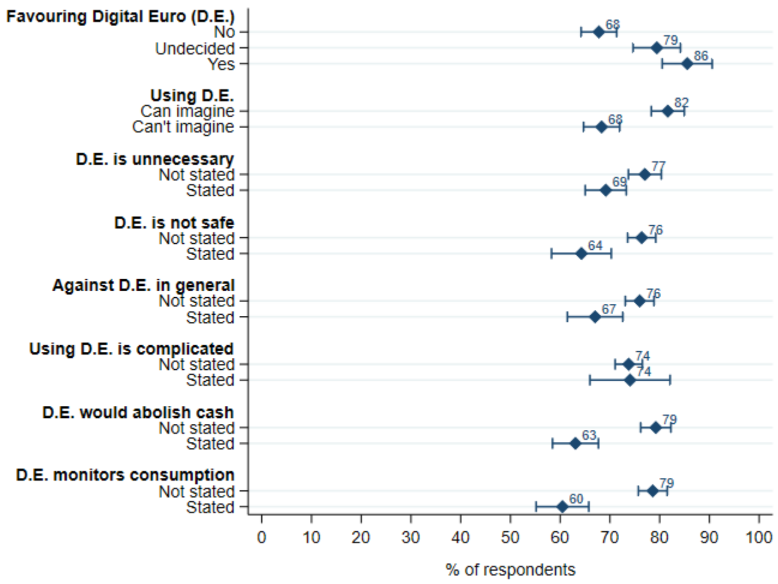

Note. N is between 2640 and 2650. Error bars represent 95% CI. All predicted consent rates per subcategories within each variable are significantly different from each other (p<0.01), aside from “Favouring D.E.” “undecided” and “yes”, and “Using D.E. is complicated” “stated” and “not stated”.

Fig. S3

Predicted consent rates to share data by individual risk and labor market affectedness – scenario 50%

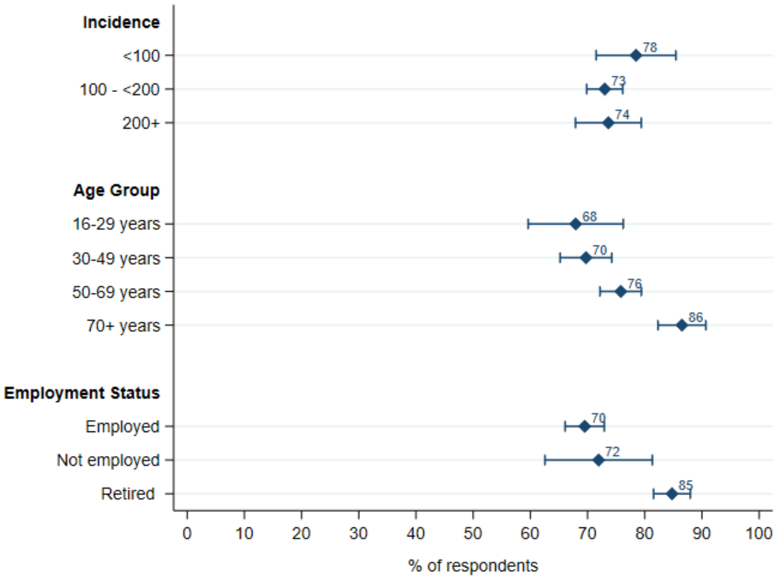

Note. N=2653 for incidence, age and employment status. Error bars represent 95% CI. Predicted significant differences: $p<0.01$: age groups “16-29 years” vs. “70+ years”, “30-49 years” vs. “70+ years”, and “50-69 years” vs. “70+ years”, “employed” vs. “retired”. $p<0.05$: age group “30-49 years” vs. “50-69 years”, “not employed” vs. “retired”.
